# Synthesis of Renewable High-Density Fuel with Vanillin and Cyclopentanone Derived from Hemicellulose

**DOI:** 10.3390/molecules28135029

**Published:** 2023-06-27

**Authors:** Wei Wang, Ling An, Chi Qian, Yanqing Li, Meiping Li, Xianzhao Shao, Xiaohui Ji, Zhizhou Li

**Affiliations:** 1 Shaanxi Key Laboratory of Catalysis, School of Chemistry and Environment Science, Shaanxi University of Technology, No. 1 East One Ring Road, Hanzhong 723001, China; wangwei@snut.edu.cn (W.W.); anling202425@163.com (L.A.); qianchi0720@163.com (C.Q.); lyq1690109355@163.com (Y.L.); xianzhaoshao@snut.edu.cn (X.S.); slgjxh@163.com (X.J.); lizhizhou136@sina.com (Z.L.); 2 College of Life Science, Shanxi University, Taiyuan 030006, China

**Keywords:** high-density fuel, vanillin, cyclopentanone, aldol condensation, hydrodeoxygenation

## Abstract

1,3-bis(cyclohexylmethyl)cyclopentane, a renewable high-density fuel, was first produced in a high overall carbon yield (79.5%) with vanillin and cyclopentanone, which can be derived from biomass. The synthetic route used in this work contains two steps. In the first step, 2,5-bis(4-hydroxy-3-methoxybenzylidene)cyclopentanone was synthesized by aldol condensation of vanillin and cyclopentanone under the catalysis of sulphuric acid. Over the optimized condensation, a high carbon yield (82.6%) of 2,5-bis(4-hydroxy-3-methoxybenzylidene) cyclopentanone was achieved at 80 ºC. In the second step, 2,5-bis(4-hydroxy-3-methoxybenzylidene) cyclopentanone was hydrodeoxygenated over the Pd/HY catalyst in cyclohexane as solvent. High carbon yields of 1,3-bis(cyclohexylmethyl)cyclopentane (96.2%) was obtained. The polycycloalkane mixture as obtained has a density of 0.943 g mL^−1^ and a freezing point of −35 °C. It can be blended into conventional high-density fuels (e.g., JP-10) for rockets and missile propulsion as a potential application.

## 1. Introduction

The rapid consumption of fossil energy, and environmental problems, have attracted more and more attention. The production of renewable chemicals and liquid fuels [1,2,3] from biomass lignocellulose is fascinating. Multi-cyclic hydrocarbons have higher densities and volumetric heating values compared with non-naphthenic and mono-cyclic hydrocarbons. In real applications, polycycloalkanes can be used to increase the range, payload and/or velocity of aircraft without increasing the volume of the oil tank. The currently used high-density fuels (for example JP-10, RJ-5, RJ-7, etc.) are derived from fossil energy by the Diels−Alder reactions of cyclopentadiene (or methyl-cyclopentadiene) followed by isomerization and hydrogenation [4,5]. The high-density fuel precursors of tricyclic and bicyclic compounds were synthesized by Diels−Alder reactions of isoprene and p-benzoquinone [6,7]. 

Bio-JP-10 fuel has been synthesized with furfuryl alcohol on an industrial scale with agricultural and forestry residues [8]. Methylcyclopentadiene dimers were produced from linalool [9]. High density fuels were synthesized by terpene [10], pinenes [11,12,13] and sesquiterpanes [14]. Several synthetic routes for renewable high-density fuels have been developed using cyclohexanone, cyclohexanol, phenol, anisole and guaiacol as feedstocks [15,16,17]. Other synthetic methods for high-energy density fuels have been reported using cyclohexanol [18,19], cyclopentanone [20,21,22], cellulose [23] and isophorone [24] as feedstocks. However, to realize large-scale industrial applications in the future, more new methods for synthesizing high-density liquid fuels will be developed. 

Cyclopentanone is a lignocellulose platform compound, which can be acquired by hydrogenation and rearrangement of furfural [25,26,27]. Vanillin is prepared through the oxidation of kraft lignin, alkali lignin, lignosulfonate, or two enzymatic lignins. The inter-unit bond of lignin had a significant effect on the yield of vanillin [28]. The more the β-*O*-4 bonds in lignin, the higher the yield of the corresponding aldehyde. The use of lignin with high β-*O*-4 bond content can significantly improve the yield of vanillin [29,30]. Vanillin was produced by partial photocatalytic oxidation of ferulic acid in an aqueous solution at ambient temperature. A photocatalytic membrane reactor was obtained by effective coupling of dialysis and photocatalytic reaction. The total amount of vanillin produced by the reaction of ferulic acid aqueous solution in the reactor for 5 h is more than that in the photocatalytic reactor without dialysis [31]. A new method for the synthesis of vanillin by a three-step two-enzyme cascade sequence was reported. Bacillus megaterium CYP102A1 catalyzes the conversion of substrate 3-methyl anisole to vanillin via inter-mediate 4-methyl guaiacol. The vanillin alcohol oxidase variant was selected to catalyze the conversion of vanillin alcohol to vanillin [32]. Vanillin and cyclohexanone undergo aldol condensation reaction and hydrodeoxygenation to obtain polycyclic alkanes of C_13_ [33]. Polycyclic alkanes of C_12_ were obtained through the aldol condensation reaction of vanillin and cyclopentanone, followed by hydrodeoxygenation [34]. 

As mentioned in the review, many methods for preparing high-density liquid fuels from biomass derivatives through carbon-carbon coupling and hydrodeoxygenation have been reported. However, due to the limited quantity of raw materials and high production costs, these methods are still in the laboratory stage and cannot be applied in industrial applications. Therefore, the continuous development of new methods for synthesizing high-density liquid fuels using biomass and its derivatives remains a current research hotspot. At the same time, the route for the synthesis of C_19_ polycyclic alkanes from vanillin and cyclopentanone via aldol condensation and hydrodeoxygenation has not been reported. Therefore, in this work, 1,3-bis(cyclohexylmethyl)cyclopentane, a C_19_ polycycloalkane with a density of 0.943 g mL^−1^ and a freezing point of −35 °C, was obtained in an overall carbon yield of ~79.5% with vanillin and cyclopentanone. According to our review, this density is high. In future applications, it can be used as fuel or an additive for rockets and missile propulsion.

## 2. Results and Discussion

**Scheme 1:**
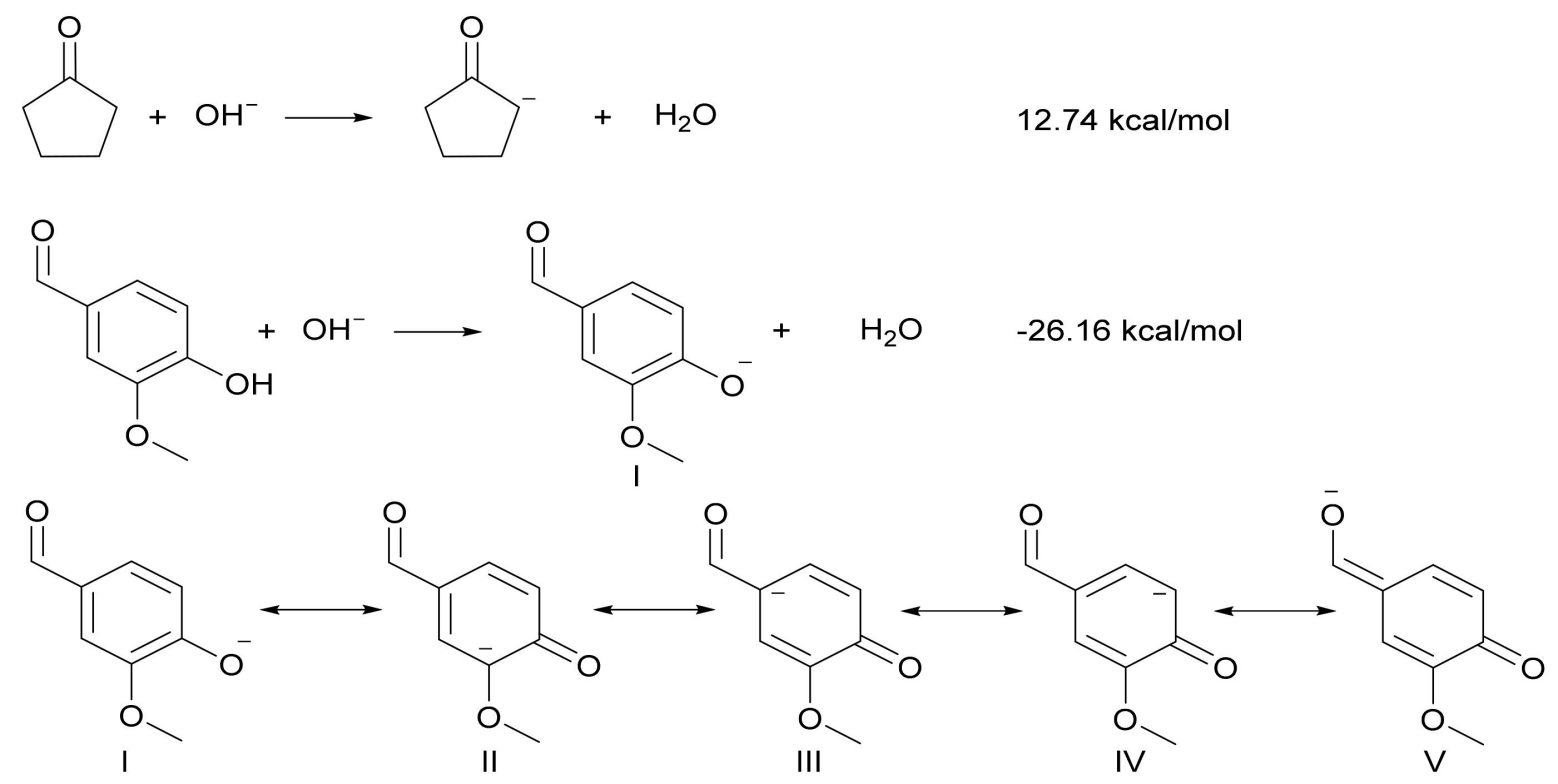
The reaction of cyclopentanone with vanillin under an alkaline catalyst.

**Scheme 2:**
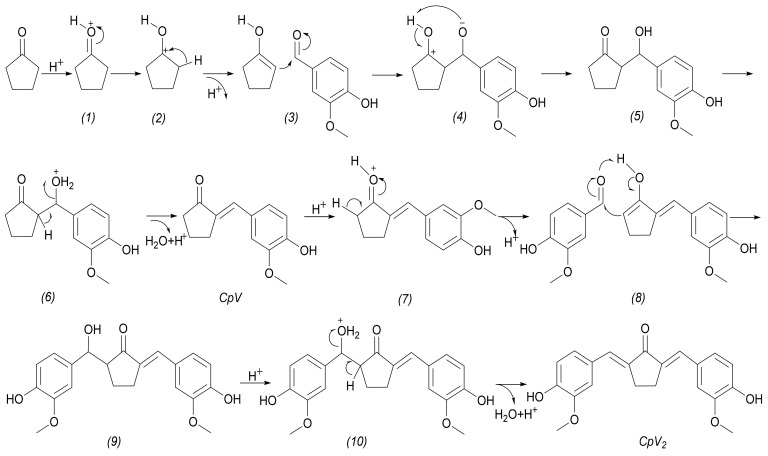
The reaction of cyclopentanone with vanillin under an acidic catalyst.

Synthesis of 2,5-bis(4-hydroxy-3-methoxybenzylidene)cyclopentanone. The reaction between vanillin and cyclopentanone is a typical aldol condensation reaction. For general aldol condensation reactions, both alkaline and acid catalysts can be used for catalysis. The reaction of cyclopentanone with vanillin over a basic catalyst is shown in Scheme 1. However, we found that the aldol condensation of vanillin and cyclopentanone could not be carried out under an alkaline catalyst. This is because the gibbs-free energy of vanillin and cyclopentanone with OH^−^ are−26.16 kcal/mol and 12.74 kcal/mol, respectively, when calculated to find the reaction of cyclopentanone with vanillin under basic catalyst by the Density Functional Theory (DFT). This indicates that under alkaline conditions, the oxygen anion of vanillin is higher than that of cyclopentanone α, and carbanion is easier to form. To our knowledge, the phenolic hydroxyl groups in vanillin are somewhat acidic (Phenol, pKa = 9.99; 4-Hydroxybenzaldehyde, pKa = 7.61), which is consistent with the DFT. The oxygen-negative ion (I) formed by vanillin under alkaline conditions can form four resonance formulations (II, III, IV and V). Due to the inability to form cyclopentanone carbon anions in the presence of alkaline catalysts, it is impossible to form aldol condensation products. Therefore, the aldol condensation reaction of vanillin with cyclopentanone can only be carried out under the acid catalyst. The pathway for the acid-catalyzed aldol condensation reaction of vanillin and cyclopentanone is shown in Scheme 2. The cyclopentanone reacts with one molecule of vanillin to produce 2-(4-hydroxy-3-methoxybenzylidene)cyclopentanone (CpV). The reaction of cyclopentanone with two molecules of vanillin produces 2,5-bis(4-hydroxy-3-methoxybenzylidene)cyclopentanone (CpV_2_).

**Figure 1:**
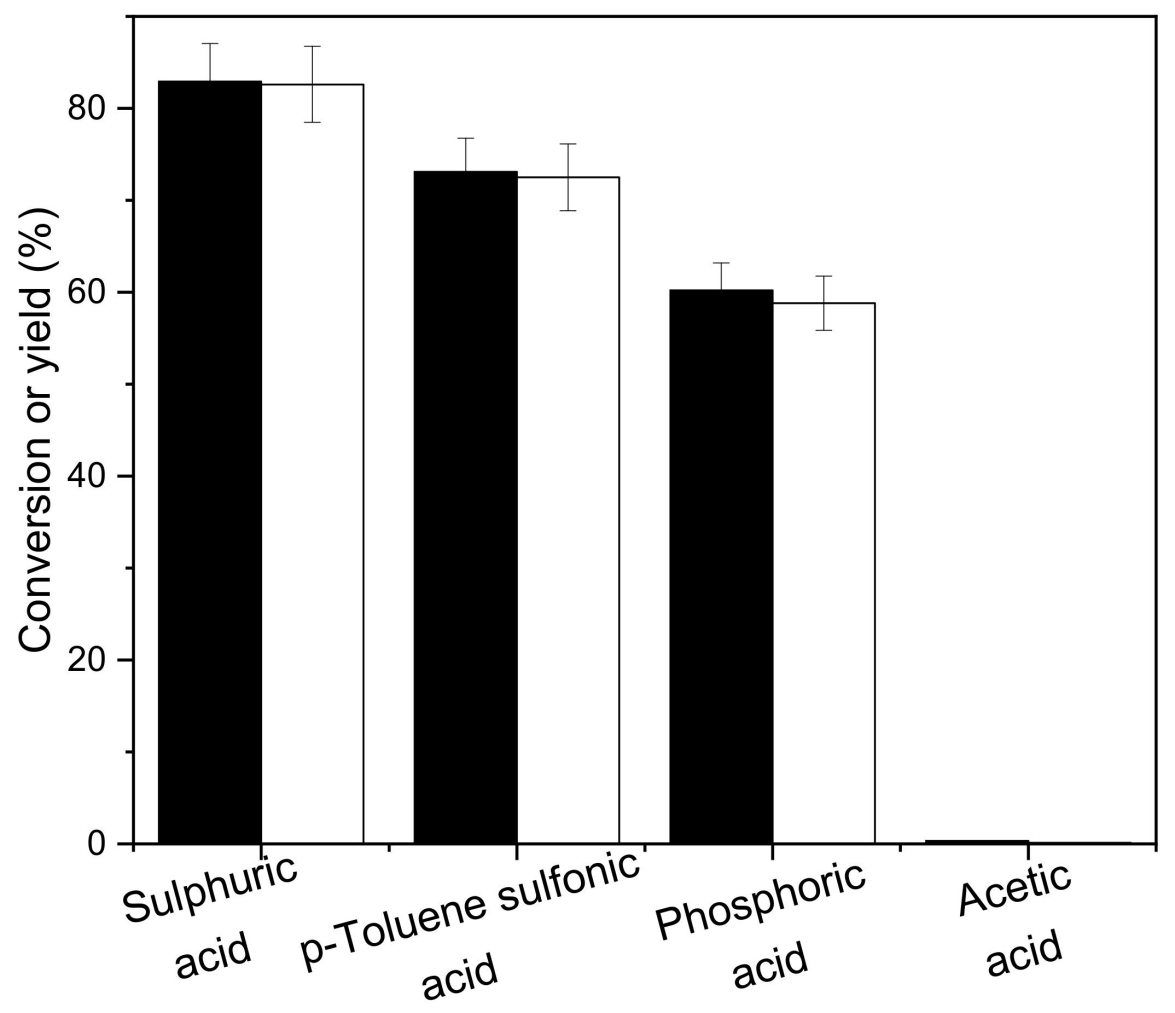
Conversions of cyclopentanone (black bars) and the carbon yields of CpV*_2_* (white bars) under the catalysis of different acids. Reaction conditions: 80 °C, 1 h; 0.84 g cyclopentanone, 3.04 g vanillin, 0.10 g concentrated sulfuric acid and 10 mL ethanol.

The synthesis of 2,5-bis(4-hydroxy-3-methoxybenzylidene)cyclopentanone was used as the target product. As shown in Figures S1 and S2 of the Supplementary Materials, the products were characterized by nuclear magnetic resonance and mass spectrometry. The influence of various catalysts on the aldol condensation of vanillin and cyclopentanone was investigated. The experimental results in Figure 1 show that the activity sequence of aldol condensation reaction of vanillin and cyclopentanone is from high to low on the catalysts of concentrated sulfuric acid, p-toluenesulfonic acid, phosphoric acid and acetic acid. The catalyst with the highest activity is concentrated sulfuric acid. The conversion of cyclopentanone is 82.9%, and the yield of 2,5-bis(4-hydroxy-3-methoxybenzylidene)cyclopentanone, is 82.6%. The next one with higher catalytic activity is p-toluenesulfonic acid. Cyclopentanone is converted to 73.1%, and the yield of 2,5-bis(4-hydroxy-3-methoxybenzylidene)cyclopentanone is 72.5%. The conversion of cyclopentanone was 60.2% under phosphoric acid catalysis, while the yield of 2,5-bis(4-hydroxy-3-methoxybenzylidene)cyclopentanone was 58.8%. Acetic acid is the least acidic of the three acids, and it scarcely combines with vanillin and cyclopentanone in the aldol condensation reaction. As a result, the catalyst was chosen to be concentrated sulphuric acid. 

**Figure 2:**
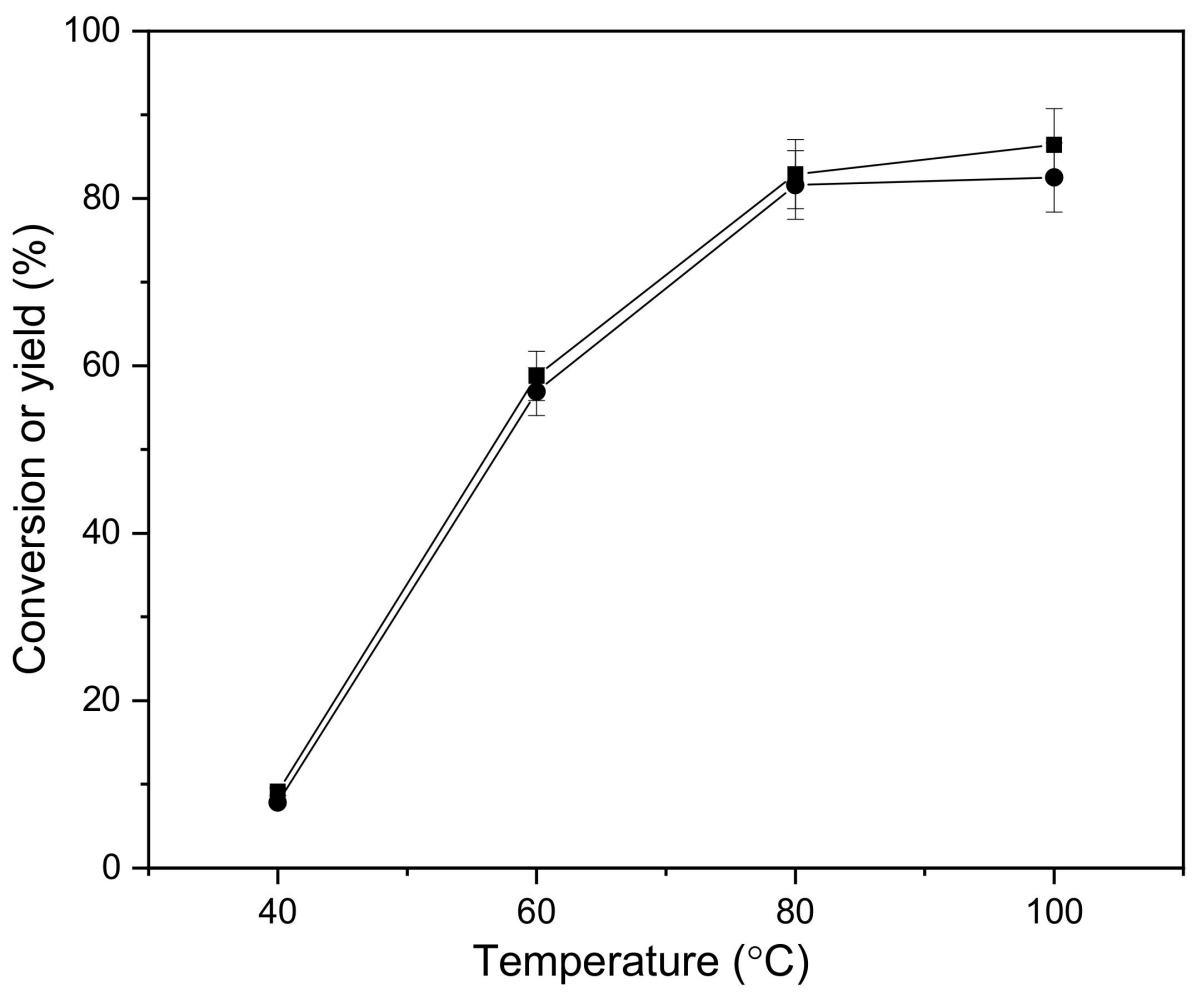
Conversion of cyclopentanone (■) and the carbon yield of CpV_2_ (●) as a function of reaction temperature. Reaction conditions: 8 h; 0.84 g cyclopentanone, 3.04 g vanillin, 0.10 g concentrated sulfuric acid and 10 mL ethanol.

Figure 2 depicts the influence of temperatures on the aldol condensation of cyclopentanone and vanillin. In the range of temperature from 40 °C to 100 °C, and with the increase of temperature, the conversion of cyclopentanone and the yield of 2,5-bis(4-hydroxy-3-methoxybenzylidene)cyclopentanone increased first, and then tended to be stable. The yield of 2,5-bis(4-hydroxy-3-methoxybenzylidene)cyclopentanone peaked at 80 °C, after which it stabilized, meaning it reached its maximum.

**Figure 3:**
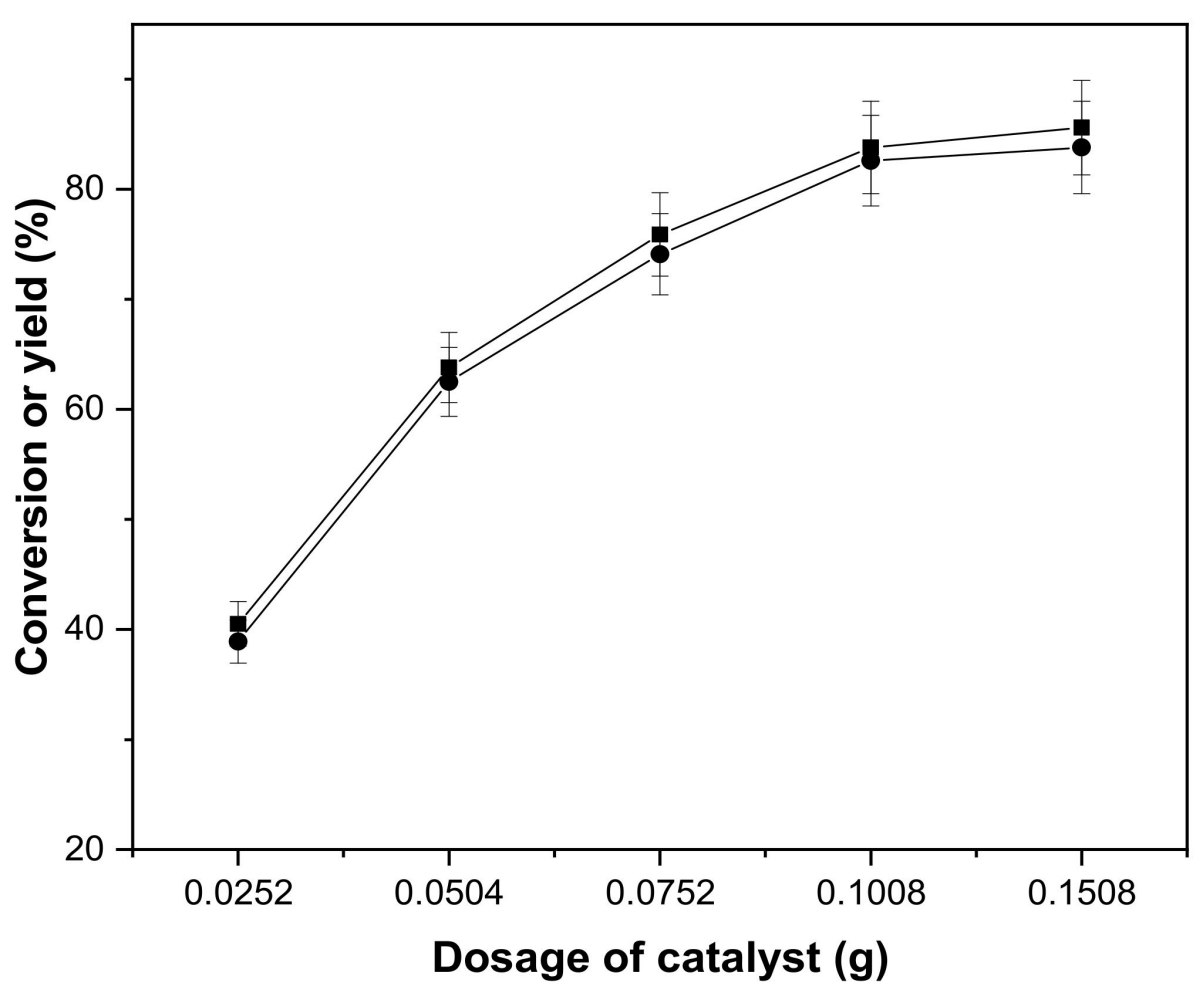
Conversion of cyclopentanone (■) and the carbon yield of CpV_2_ (●) as a function of catalyst dosage. Reaction conditions: 80 °C, 8 h; 0.84 g cyclopentanone, 3.04 g vanillin and 10 mL ethanol.

The impact of catalyst dose on aldol condensation of the cyclopentanone and vanillin is depicted in Figure 3. Within the range of 0.0250 g to 0.1500 g of concentrated sulphuric acid, the conversion of cyclopentanone and the yield of 2,5-bis(4-hydroxy-3-methoxybenzylidene)cyclopentanone exhibited a rising and then steady trend. At 0.1008 g, the greatest yield of 2,5-bis(4-hydroxy-3-methoxy benzylidene)cyclopentanone was obtained. A tiny amount of concentrated sulphuric acid was added, which resulted in a smaller acid center and reduced output. As the quantity of concentrated sulphuric acid grows, the acid center expands and the cyclopentanone activity expands, enabling easier and faster aldol condensation. 

**Figure 4:**
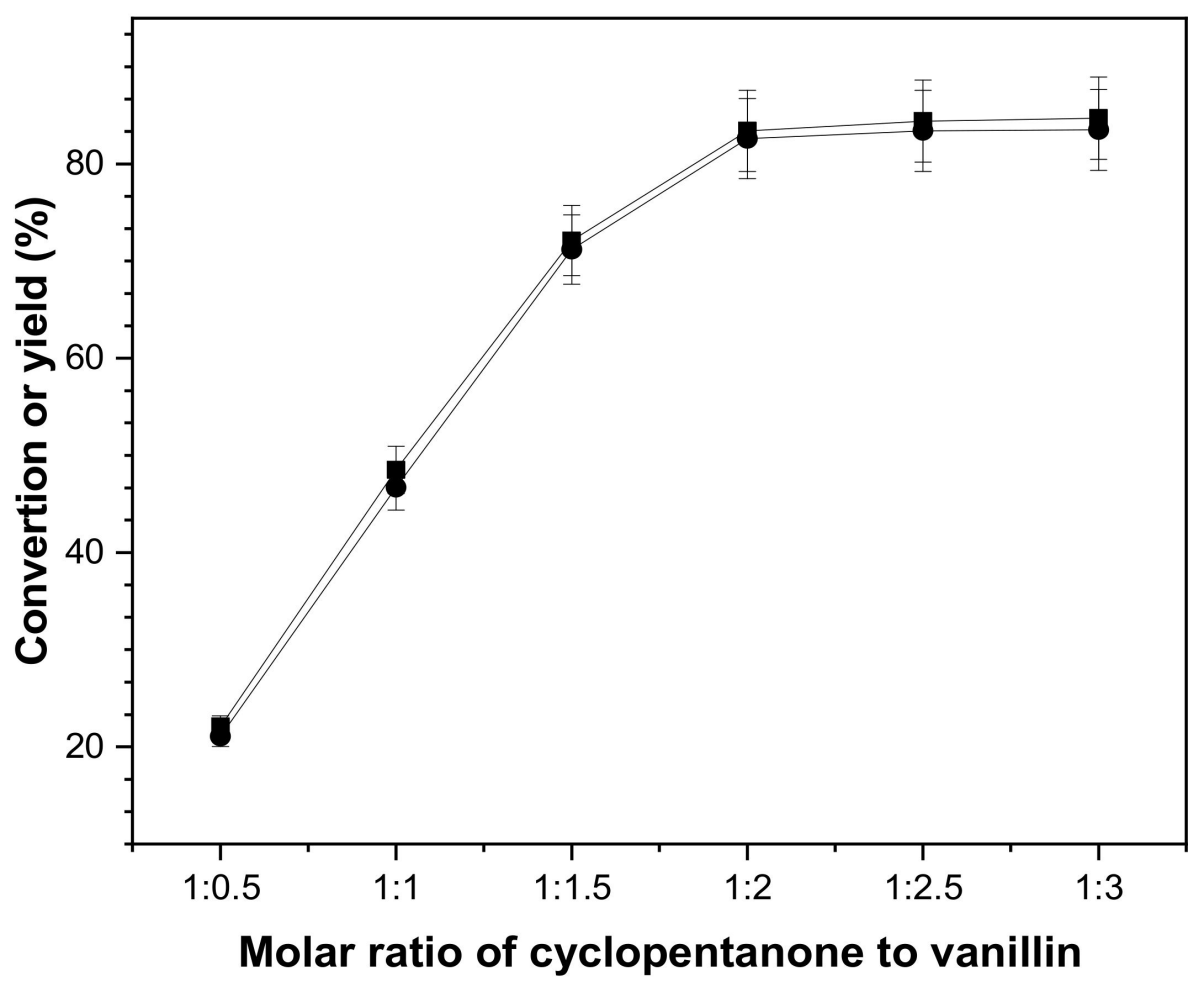
Conversion of cyclopentanone (■) and the carbon yield of CpV_2_ (●) as the molar ratio of cyclopentanone to vanillin. Reaction conditions: 80 °C, 8 h; 0.84 g cyclopentanone, 0.10 g concentrated sulfuric acid and 10 mL ethanol.

Figure 4 illustrates the influence of the cyclopentanone to vanillin molar ratio on the aldol condensation reaction of cyclopentanone and vanillin, which demonstrates that cyclopentanone conversion and yield of 2,5-bis(4-hydroxy-3-methoxy benzylidene)cyclopentanone rose and subsequently stabilized in the range of 1:0.5 to 1:3. The conversion of cyclopentanone and yield of 2,5-bis(4-hydroxy-3-methoxy benzylidene)cyclopentanone were increasing and then stabilizing. The yield of 2,5-bis(4-hydroxy-3-methoxy benzylidene)cyclopentanone was maximized when the ratio of cyclopentanone and vanillin were 1:2. 

**Figure 5:**
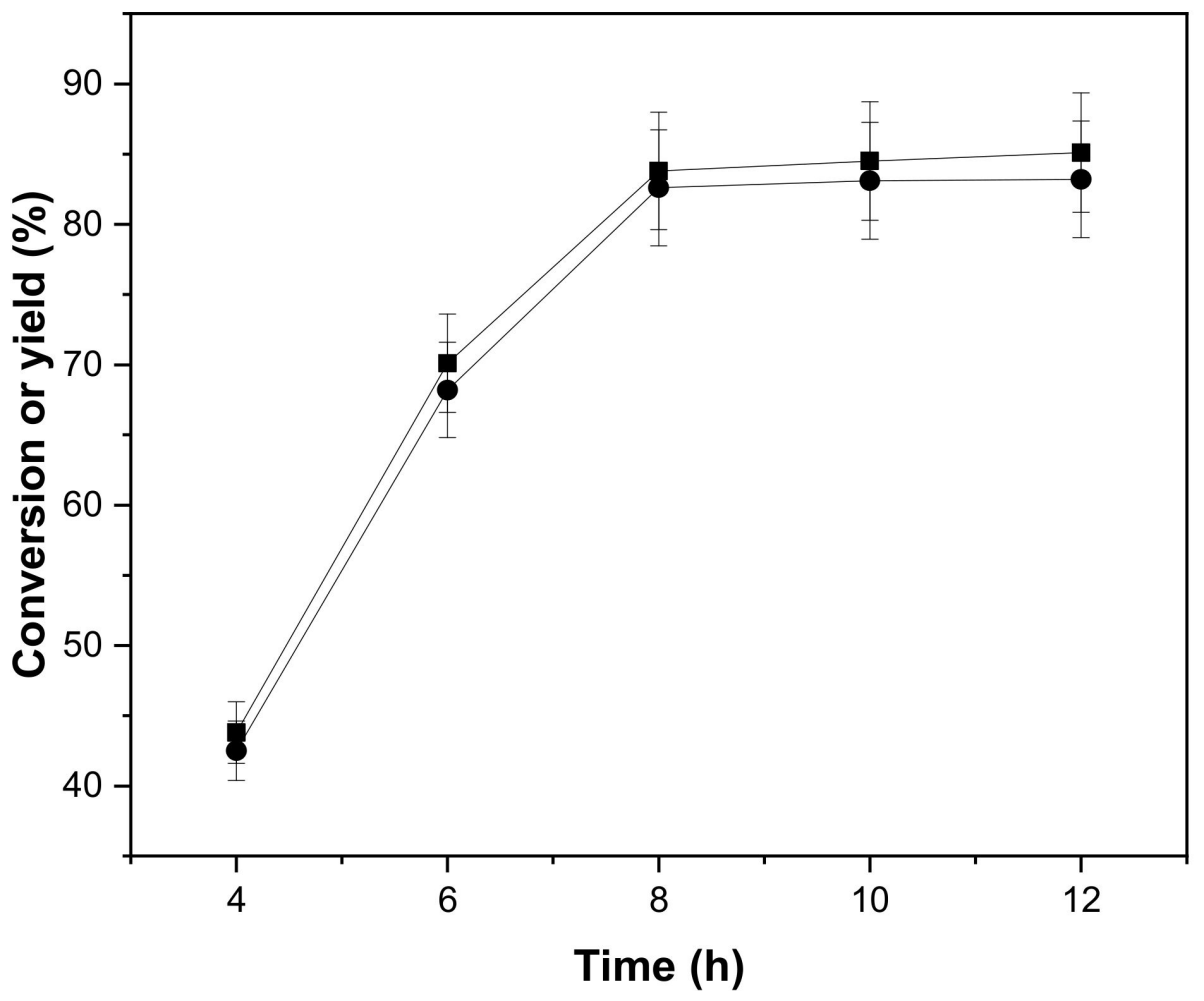
Conversion of cyclopentanone (■) and the carbon yield of CpV_2_ (●) as a function of reaction time. Reaction conditions: 80 °C; 0.84 g cyclopentanone, 3.04 g vanillin, 0.10 g concentrated sulfuric acid and 10 mL ethanol.

The aldol condensation of cyclopentanone with vanillin was also affected by reaction times, as shown in Figure 5. The conversion of cyclopentanone and the yield of 2,5-bis(4-hydroxy-3-methoxybenzylidene) cyclopentanone increased first and then stabilized within 4 h to 12 h. At 8 h, the yield of 2,5-bis(4-hydroxy-3-methoxybenzylidene)cyclopentanone reached the maximum, so the optimal reaction time was 8 h. With the extension of reaction time, the quantity of reactants decreased, and the number of products gradually increased. After a period of reaction, the aldol condensation reached a dynamic equilibrium, and the yield of 2,5-bis(4-hydroxy-3-methoxybenzylidene) cyclopentanone did not change.

Based on the above single-factor experimental optimization results, the conversion of cyclopentanone is 82.9%, and the yield of 2,5-bis(4-hydroxy-3-methoxybenzylidene)cyclopentanone is 82.6% under the optimized conditions: the molar ratio of cyclopentanone to Vanillin is 1:2, the reaction temperature is 80 °C, the amount of concentrated sulfuric acid is 0.1008 g, 10 mL ethanol, and the time is 8 h.

**Figure 6:**
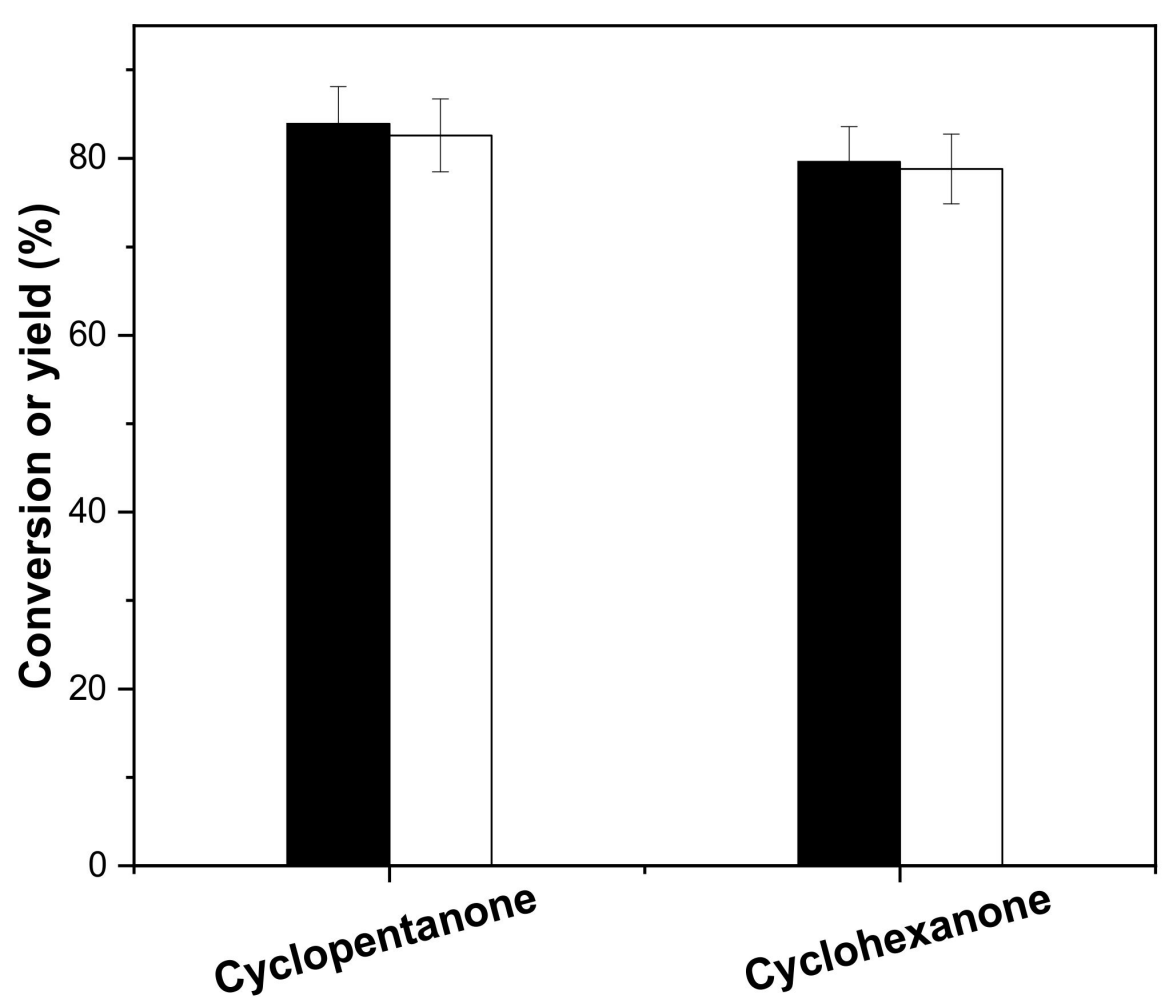
Conversion (black bars) of cyclopentanone (cyclohexanone) and the carbon yield (white bars) of CpV_2_ (and ChV_2_) as a function of reaction time. Reaction conditions: 80 °C; 0.84 g cyclopentanone (0.98 g cyclohexanone), 3.04 g vanillin, 0.10 g concentrated sulfuric acid and 10 mL ethanol.

Under the optimized conditions, aldol condensation of cyclohexanone and vanillin was implemented, and the results are shown in Figure 6. The conversion of cyclohexanone is 79.6%, and the yield of 2,6-bis(4-hydroxy-3-methoxybenzylidene)cyclohexanone (ChV_2_) is 78.8%. ChV_2_ was characterized by nuclear magnetic resonance and mass spectrometry as shown in Figures S3 and S4.

**Scheme 3:**
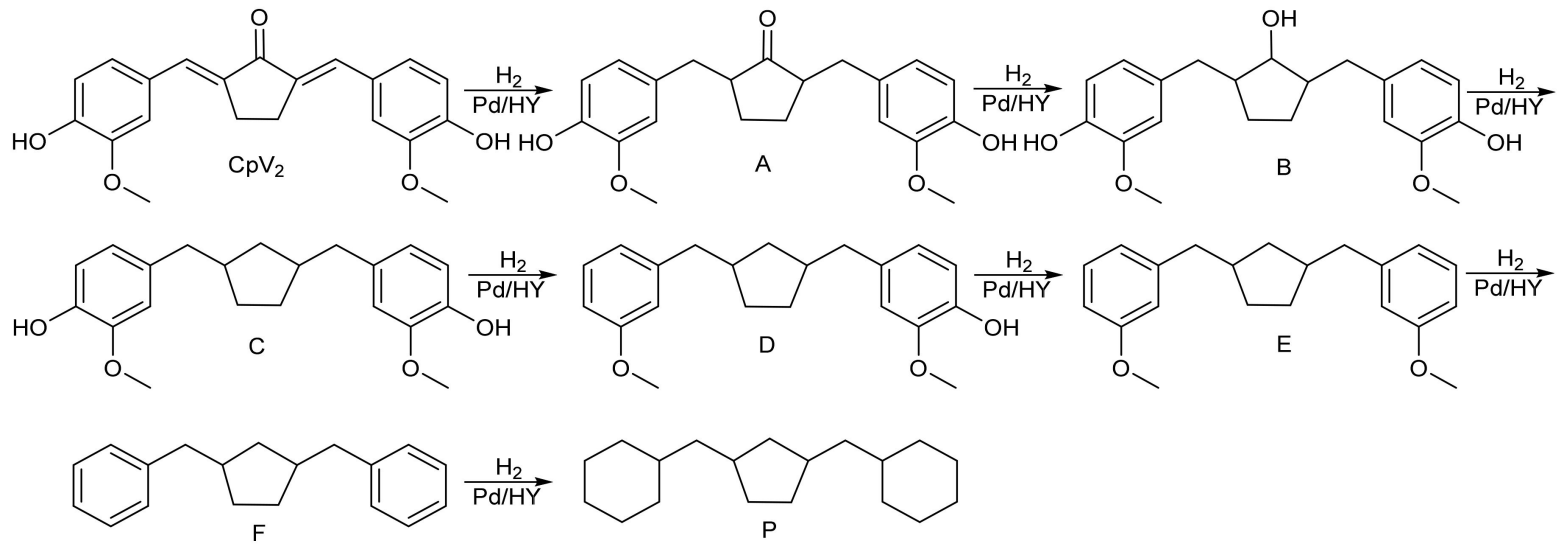
Reaction pathway for the generation of different products from the hydrodeoxygenation of CpV_2_ over the 5 wt% Pd/HY catalyst.

The objective of this work is to synthesize biomass liquid fuel. Considering that the 2,5-bis(4-hydroxy-3-methoxybenzylidene) cyclopentanone contains benzene rings, C=C double bonds, C=O double bonds, hydroxyl groups, and methoxy group functional groups, the hydrodeoxygenation catalyst needs a bifunctional catalyst containing active metal and solid acid. A pathway for hydrodeoxygenation of 2,5-bis(4-hydroxy-3-methoxybenzylidene) cyclopentanone can be seen in Scheme 3.

The hydrodeoxygenation product of 2,5-bis(4-hydroxy-3-methoxybenzylidene)cyclopentanone (CpV_2_) was analyzed by GC-MS, as shown in Figure S5. The C=C double bond in CpV_2_ is first hydrogenated, followed by the hydrogenation of the C=O double bond to 4,4′-((2-hydroxy Cyclopentane-1,3-diyl) bis (methylene)) bis (2-methoxy phenol) (B). B continues to be hydrogenated, and the hydroxyl group on the cyclopentanol in B is removed to generate 4,4‘-(cyclopentane-1,3-diylbis (methylene)) bis (2-Methoxy phenol) (C). C is further hydrogenated, and the hydroxyl group on the phenol in C is removed to generate 2-methoxy-4-((3-(trimethoxybenzyl) cyclopentyl) methyl) phenol (D) and 1,3-bis (3-methyloxybenzyl) cyclopentane (E). Through the hydrogenolysis of methyl phenyl ether in E, 1,3-dibenzyl cyclopentane (F) is obtained, and then the benzene ring in F is further hydrogenated to obtain the target of 1,3-bis (cyclohexylmethyl) cyclopentane (P).

**Figure 7:**
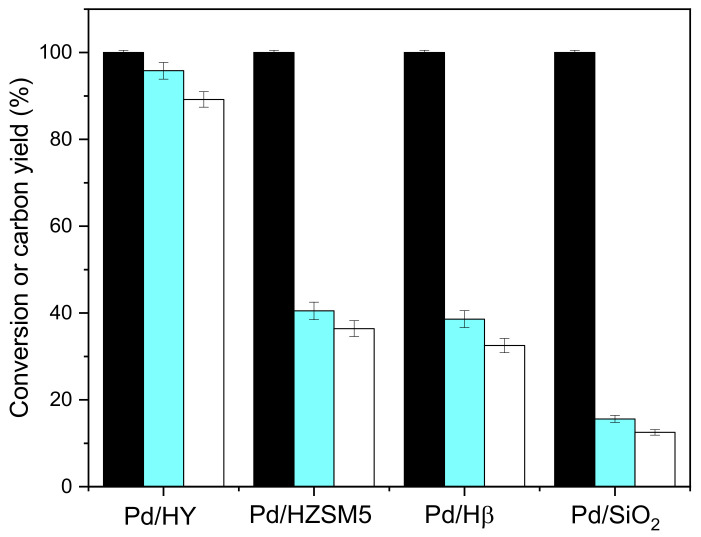
Conversion of CpV_2_ (black bars), carbon yields of C_9_–C_19_ diesel range alkanes (blue bars), and C_19_ alkanes (white bars) over palladium supported on different solid acid catalysts. Reaction conditions: 260 °C, 24 h; 1.0 g CpV_2_, 4 MPa H_2_, 0.1 g Pd/M (M = HY, HZSM5, Hβ and SiO_2_) catalyst and 40 mL cyclohexane were used for each test.

The hydrodeoxygenation of CpV_2_ is carried out on different solid acid catalysts supported on palladium. The results are shown in Figure 7. The hydrodeoxygenation activity is in the order: Pd/HY > Pd/Hβ > Pd/HZSM5 > Pd/SiO_2_. This activity sequence was in good agreement with the acidity of the carrier (-H° of HY, Hβ and HZSM5 were 4.4, 4.4–5.7 and 5.6–5.7, respectively). Under the catalysis of Pd/HY, the yield of 1,3-bis (cyclohexylmethyl)cyclopentane was 89.2%, and the yield of C_9_–C_19_ diesel ranges was 95.8%. Therefore, the selected catalyst carrier is HY.

**Figure 8:**
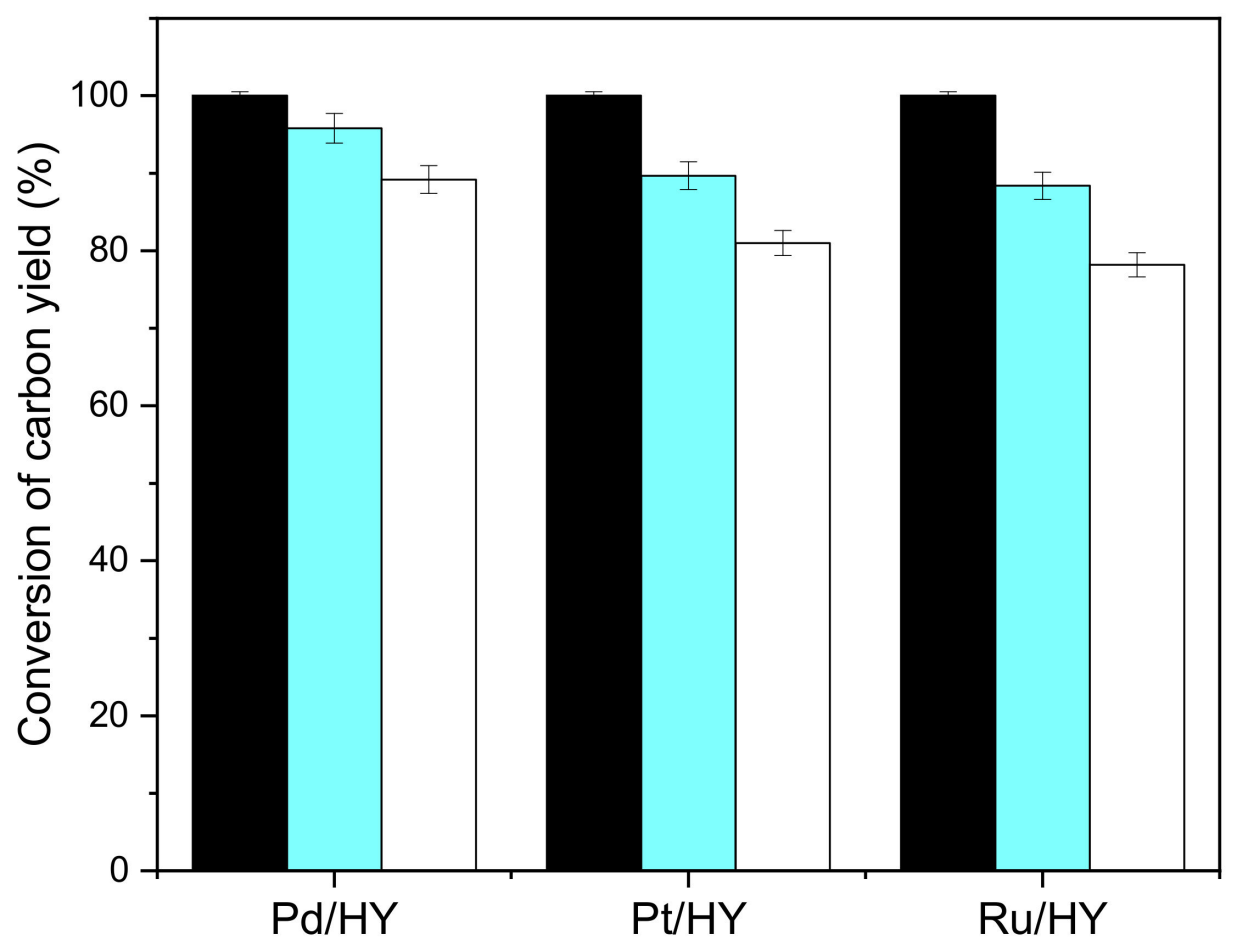
Conversion of CpV_2_(black bars), carbon yields of C_9_–C_19_ diesel range alkanes (blue bars), and C_19_ alkanes (white bars) over different metals supported on HY catalysts. Reaction conditions: 260 °C, 24 h; 1.0 g CpV_2_, 4 MPa H_2_, 0.1 g C/HY (C = Pd, Pt, Ru) catalyst and 40 mL cyclohexane were used for each test.

At the same time, a study was conducted to determine catalytic activity of different metals on the HY carrier affecti**ng** the hydrodeoxygenation of CpV_2_. The experimental results are shown in Figure 8. The activity order of the catalyst for hydrodeoxygenation of CpV_2_ is Pd/HY > Pt/HY > Ru/HY. Pd was chosen as the active metal, has better hydrogenation activity and selectivity than Pt. 

**Figure 9:**
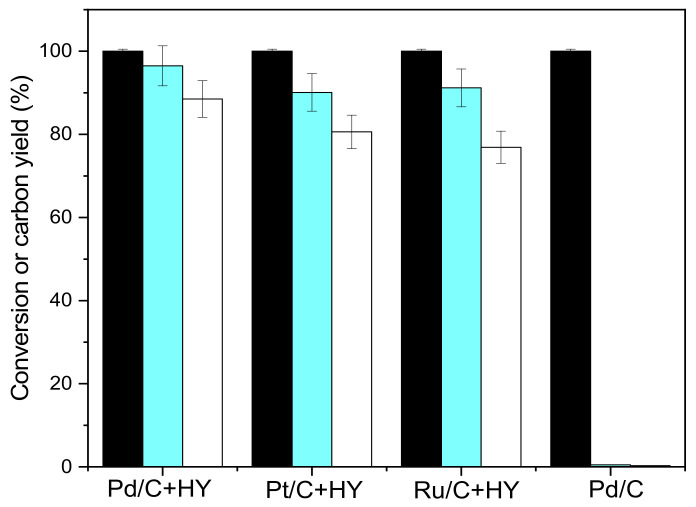
Conversion of CpV_2_ (black bars), carbon yields of C_9_–C_19_ diesel range alkanes (blue bars), and C_19_ alkanes (white bars) over different catalysts. Reaction conditions: 260 °C, 24 h; 1.0 g CpV_2_, 4 MPa H_2_, 0.1 g Pd/+M (M = HY, HZSM5, Hβ) catalyst and 40 mL cyclohexane were used for each test.

In this paper, catalysts of Pd/C + HY, Pt/C + HY and Ru/C + HY were also prepared by a grinding method, and the obtained catalysts were used to catalyze the hydrodeoxygenation of CpV_2_. The reaction results are shown in Figure 9. The activity sequences of Pd/C + HY, Pt/C + HY and Ru/C + HY were consistent with those of Pd/HY, Pt/HY and Ru/HY. Under the catalysis of Pd/C + HY, the yield of 1,3-bis (cyclohexylmethyl)cyclopentane was 88.5%, and the yield of the C_9_–C_19_ diesel ranges was 96.5%. Pd/C can only hydrogenate the C=C and C=O double bonds in CpV_2_ to generate 4,4′-((2-hydroxycyclopentane-1,3-diyl)bis(methylene))bis(2-methoxyphenol). 

Under the optimized conditions of CpV_2_ hydrodeoxygenation, 2,6-bis(4-hydroxy-3-methoxybenzylidene)-cyclohexanone was hydrodeoxygenated and completely converted. The hydrodeoxygenation product of 2,6-bis (4-hydroxy-3-methoxybenzylidene)cyclohexanone was analyzed by GC-MS, as shown in Figure S6. The yield of 1,3-bis (cyclohexylmethyl) cyclohexene was 89.5%, and the carbon yield of C_9_–C_19_ diesel range alkanes was 96.8%. 

The density and freezing point of mixture A and mixture B of 2,5-bis(4-hydroxy-3-methoxybenzylidene)cyclopentanone and 2,6-bis(4-hydroxy-3-methoxybenzylidene)-cyclohexanone hydrodeoxygenation were measured. The density and the freezing point of mixture A obtained in this work were 0.943 g mL^−1^ and −32 °C, respectively. The density and the freezing point of mixture B in this work were 0.946 g mL^−1^ and −35 °C, respectively. These properties are similar to those of JP-10, and the hydrogenated pinene dimers have been reported in the previous work of Harvey, Zou and their co-workers. These mixtures of alkanes can be used as high-density liquid fuels or fuel additives.

## 3. Materials and Methods

### 3.1. Material

Vanillin (99%), cyclopentanone (98%), cyclohexanone (99%), ethanol (95%), phosphoric acid (85%) and acetic acid (99.5%) were purchased by West Asian reagents. Methanol (99.5%) was purchased from Aladdin Reagent Plant (Shanghai, Country). Concentrated sulfuric acid (98%) and p-toluenesulfonic acid (98%) were obtained by Beijing Chemical Reagent Factory. PdCl_2_, RuCl_3_∙3H_2_O, H_2_PtCl_6_∙3H_2_O were purchased by Tianjin FengChuan Chemical Reagent Technology Co., Ltd. (Tianjin, Country) Pd/C, Pt/C and Ru/C were supplied by Aladdin Reagent Plant. HY, H-β, HZSM5 and SiO_2_ (99%) were collected by catalyst plant of Nanjing University. The chemicals mentioned above are all made in China.

### 3.2. Catalyst Preparation

The Pd/HY catalyst used for hydrodeoxygenation of CpV_2_ was prepared by the incipient wetness impregnation of HY with an aqueous solution of PdCl_2_. The catalyst precursors obtained were dried at 80 °C for 24 h and then reduced by H_2_ (160 mL min^−1^ g_cat_^−1^) at 400 °C for 2 h. After cooling in H_2_ to room temperature, the catalysts were passivated with 1 vol.% O_2_ in N_2_. To facilitate comparison, the metal content in each catalyst was fixed at 5% by weight (denoted as 5 wt.%). Preparation of Pt/HY, Ru/HY, Pd/HZSM5, Pd/Hβ and Pd/SiO_2_ catalysts are the same as the above method.

### 3.3. Activity Test

The direct synthesis of 2,5-bis(4-hydroxy-3-methoxybenzylidene)cyclopentanone by the aldol condensation of cyclopentanone and vanillin was conducted in a stainless steel batch reactor with Teflon. For each test, 0.84 g cyclopentanone, 3.04 g vanillin, 0.10 g sulphuric acid and 10 mL ethanol were utilized. Before the test, the stainless-steel batch reactor was purged by nitrogen two times. The mixture of catalyst and reactant was stirred at 80 °C for 8 h. Subsequently, the stainless-steel batch reactor was cooled to room temperature with water, and the reaction mixture was quenched with 20 mL of ice water. The reaction mixture was filtered and the solid was washed twice with 10 mL of ethanol (10 mL × 2). The yellow solid obtained was 2,5-bis(4-hydroxy-3-methoxybenzylidene)cyclopentanone. The yield of 2,5-bis(4-hydroxy-3-methoxybenzylidene)cyclopentanone was calculated. The filtrate was analyzed by gas chromatograph (GC) of Agilent 7890B, which is equipped with a HP-INNOWAX capillary column (30 m, 0.25 mm ID, 0.5 mm film) and a flame ionization detector (FID). The oven temperature was held at 40 °C for 2 min, increased to 280 °C at a rate of 15 °C min**^−^**^1^, and stayed at that temperature for 5 min. Nitrogen was used as the carrier gas at a flow rate of 1.5 mL min^−1^. The conversion of cyclopentanone was determined by gas chromatography. The conversion of cyclopentanone was calculated according to the following formulas:

$$conversion=\frac{The\;amount\;of\;consumed\;cyclopentanone\left(mol\right)}{The\;amount\;of\;cyclopentanone\;input\left(mol\right)}\times 100\%$$

The aldol condensation of cyclohexanone and vanillin to 2,6-bis(4-hydroxy-3-methoxy benzylidene) cyclohexanone was conducted in a stainless-steel batch reactor with Teflon. The reaction conditions, post-treatment and determination methods were the same as those of 2,5-Bis(4-hydroxy-3-methoxybenzylidene)cyclopentanone. The yield of 2,5-Bis(4-hydroxy-3-methoxybenzylidene)cyclopentanone was calculated according to the following formulas:

$$yield\left(\%\right)=\frac{The\;amount\;of\;generates\;2,5-Bis\left(4-hydroxy-3-methoxybenzylidene\right)cyclopentanone\left(mol\right)}{The\;amount\;of\;theoretically\;generates\;2,5-Bis\left(4-hydroxy-3-methoxybenzylidene\right)cyclopentanone\left(mol\right)}\times 100\%$$

The HDO of the CpV_2_ was conducted at a 100 mL high-pressure reactor. Typically, 1.0 g 2,5-bis(4-hydroxy-3-methoxybenzylidene) cyclopentanone, 0.10 g Pd/HY, 0.10 g n-tridecane as internal standard and 40.0 mL cyclohexane were vigorously stirred at 260 ºC and ~4 MPa for 24 h. After the reaction was completed, we cooled it to room temperature with water, centrifuged the reaction mixture (10,000 rpm) for 2 min, took samples for gas chromatography, and calculated the yield. The carbon yields of different alkanes in the HDO process were calculated according to the following formulas:

$$carbon\;yield\left(\%\right)=\frac{Total\;carbon\;of\;C_{9}-C_{19}\;alkanes\;in\;the\;liquid\;products}{Total\;carbon\;of\;feedstock}\times 100\%$$

$$carbon\;yield\left(\%\right)=\frac{Total\;carbon\;of\;C_{5}-C_{8}\;alkanes\;in\;the\;liquid\;products}{Total\;carbon\;of\;feedstock\;pumped\;into\;the\;reactor\;per\;unit\;time}\times 100\%$$

### 3.4. Computing Method

The density functional theory was used to verify the proposed mechanism. The RB3LYP/UB3LYP/6-311+G(d,p) basis set was used to optimize the structure of cyclopentanone and vanillin, as well as their carbon-negative ions. The frequency of the obtained molecular structure was then calculated using the above basis set to ensure that no imaginary frequency existed in the calculated structure and enthalpy of formation.

## 4. Conclusions

A new method has been developed for synthesizing high-density biomass liquid fuel. 2,5-bis(4-hydroxy-3-methoxybenzylidene)cyclopentanone was prepared from aldol condensation of vanillin and cyclopentanone by sulfuric acid. Various factors affecting aldol condensation of vanillin and cyclopentanone have been studied, including reaction temperature, catalyst dosage, cyclopentanone-vanillin molar ratio and reaction time. The yield of 2,5-bis(4-hydroxy-3-methoxybenzylidene)cyclopentanone was 82.6% under optimized conditions (80 °C, 8 h, 0.10 g sulfuric acid). Under these conditions, the aldol condensation of cyclohexanone with vanillin was extended, and the yield of 2,6-bis(4-hydroxy-3-methoxybenzylidene)cyclohexanone was 78.8%. Hydrodeoxygenation of 2,5-bis(4-hydroxy-3-methoxybenzylidene)cyclopentanone was implemented over Pd/HY, Pt/HY, Ru/HY, Pd/HZSM5, Pd/Hβ, Pd/SiO_2_, Pt/C + HY, and Ru/C + HY catalysts. At 260 °C, 4 MPa H_2_, and 24 h the conversion of 2,5-bis(4-hydroxy-3-methoxybenzylidene)cyclopentanone was 100%, and yield of 1,3-bis(cyclohexylmethyl)cyclopentane was 89.2%, and the yield of C_9_-C_19_ diesel range alkanes was 95.8% over Pd/HY. These conditions created an 89.5% yield of 1,3-bis(cyclohexylmethyl)cyclohexane and 96.8% carbon yield of C_9_–C_19_ diesel range alkane. The density and freezing point of mixture A and mixture B of 2,5-bis(4-hydroxy-3-methoxybenzylidene)cyclopentanone and 2,6-bis(4-hydroxy-3-methoxybenzylidene)hydrodeoxygenation also were tested. The densities of mixture A and mixture B were 0.943 g·mL^−^^1^ and 0.946 g·mL^−^^1^, respectively. At the same time, the freezing points of mixture A and mixture B were −32 °C and −35 °C, respectively. This is similar to the properties of JP-10. Those mixtures are suitable as high-density aviation fuels or additives. As a potential application, it can be blended into conventional high-density fuels (such as JP-10) for rockets and missile propulsion.

## Data Availability

Not applicable.

## Supplementary Materials

The following supporting information can be downloaded at: https://www.mdpi.com/article/10.3390/molecules28135029/s1, Figure S1: The ^1^HNMR and ^13^CNMR spectra of 2,5-bis(4-hydroxy-3-methoxybenzylidene)cyclopentanone; Figure S2: Mass spectra of 2,5-bis(4-hydroxy-3-methoxybenzylidene)cyclopentanone; Figrue S3: The ^1^HNMR and ^13^CNMR spectra of 2,6-bis(4-hydroxy-3-methoxybenzylidene)cyclohexanone; Figrue S4: Mass spectra of 2,6-bis(4-hydroxy-3-methoxybenzylidene)cyclohexanone; Figure S5: Gas chromatography of 2,5-bis(4-hydroxy-3-methoxybenzylidene) cyclopentanone hydrodeoxygenated product; Figure S6: Gas chromatography of 2,6-bis(4-hydroxy-3-methoxybenzylidene) cyclohexanone hydrodeoxygenated product; Table S1: Factors and Levels; Supplementary data associated with this article can be found, in the online version, at https://doi.org/10.1016/j.indcrop (accessed on 24 June 2023).

## References

[1] Fang W., Liu S., Schill L., Kubus M., Bligaard T., Riisager A. (2023). On the role of Zr to facilitate the synthesis of diesel and jet fuel range intermediates from biomass-derived carbonyl compounds over aluminum phosphate. Appl. Catal. B Environ..

[2] Wang X., Zhang C., Zhang Z., Li Q. (2022). Tuning dual active sites of Cu/CoCeOx catalysts for efficient catalytic transfer hydrogenation of 5-hydroxymethylfurfural to biofuel 2,5-dimethylfuran. Fuel.

[3] Qiu B., Tao X., Wang J., Liu Y., Li S., Chu H. (2022). Research progress in the preparation of high-quality liquid fuels and chemicals by catalytic pyrolysis of biomass: A review. Energy Convers. Manag..

[4] Chung H.S., Chen C.S.H., Kremer R.A., Boulton J.R., Burdette G.W. (1999). Recent Developments in High-Energy-Density Liquid Hydrocarbon Fuels. Energy Fuels.

[5] Zarezin D.P., Rudakova M.A., Bykov V.I., Bermeshev M.V. (2021). Metal chlorides supported on silica as efficient catalysts for selective isomerization of endo-tetrahydrodicyclopentadiene to exo-tetrahydrodicyclopentadiene for JP-10 producing. Fuel.

[6] Luo X., Lu R., Si X., Jiang H., Shi Q., Ma H., Lu F. (2022). Sustainable synthesis of high-density fuel via catalytic cascade cycloaddition reaction. J. Energy Chem..

[7] Liu C., Hu Y., Li G., Wang A., Cong Y., Wang X., Li N. (2022). Synthesis of renewable alkylated decalins with p-quinone and 2-methyl-2,4-pentanediol. Sustain. Energy Fuels.

[8] Li G., Hou B., Wang A., Xin X., Cong Y., Wang X., Li N., Zhang T. (2019). Making JP-10 Superfuel Affordable with Lignocellulosic Platform Component. Angew. Chem. Int. Ed. Engl..

[9] Meylemans H.A., Quintana R.L., Goldsmith B.R., Harvey B.G. (2011). Solvent-free conversion of linalool to methylcyclopentadiene dimers: A route to renewable high-density fuels. ChemSusChem.

[10] Meylemans H.A., Quintana R.L., Harvey B.G. (2012). Efficient conversion of pure and mixed terpene feedstocks to high density fuels. Fuel.

[11] Nie G., Zou J.J., Feng R., Zhang X., Wang L. (2014). HPW/MCM-41 catalyzed isomerization and dimerization of pure pinene and crude turpentine. Catal. Today.

[12] Zou J.J., Chang N., Zhang X., Wang L. (2012). Isomerization and Dimerization of Pinene using Al-Incorporated MCM-41 Mesoporous Materials. ChemCatChem.

[13] Harvey B.G., Wright M.E., Quintana R.L. (2010). High-Density Renewable Fuels Based on the Selective Dimerization of Pinenes. Energy Fuels.

[14] Harvey B.G., Merriman W.W., Koontz T.A. (2015). High-Density Renewable Diesel and Jet Fuels Prepared from Multicyclic Sesquiterpanes and a 1-Hexene-Derived Synthetic Paraffinic Kerosene. Energy Fuels.

[15] Nie G., Zhang X., Han P., Xie J., Pan L., Wang L., Zou J.J. (2017). Lignin-derived multi-cyclic high density biofuel by alkylation and hydrogenated intramolecular cyclization. Chem. Eng. Sci..

[16] Nie G., Zhang X., Pan L., Wang M., Zou J.J. (2018). One-pot production of branched decalins as high-density jet fuel from monocyclic alkanes and alcohols. Chem. Eng. Sci..

[17] Deng Q., Nie G., Pan L., Zou J.J., Zhang X., Wang L. (2015). Highly selective self-condensation of cyclic ketones using MOF-encapsulating phosphotungstic acid for renewable high-density fuel. Green Chem..

[18] Chen F., Li N., Yang X., Li L., Li G., Li S., Zhang T. (2016). Synthesis of High-Density Aviation Fuel with Cyclopentanol. ACS Sustain. Chem. Eng..

[19] Sheng X., Li N., Li G., Wang W., Yang J., Cong Y., Zhang T. (2015). Synthesis of high density aviation fuel with cyclopentanol derived from lignocellulose. Sci. Rep..

[20] Wang W., Li N., Li G., Li S., Wang W., Wang A., Zhang T. (2017). Synthesis of Renewable High-Density Fuel with Cyclopentanone Derived from Hemicellulose. ACS Sustain. Chem. Eng..

[21] Yang J., Li N., Li G., Wang W., Wang A., Wang X., Zhang T. (2014). Synthesis of renewable high-density fuels using cyclopentanone derived from lignocellulose. Chem. Commun..

[22] Xu J., Li N., Li G., Han F., Wang A., Cong Y., Wang X., Zhang T. (2018). Synthesis of high-density aviation fuels with methyl benzaldehyde and cyclohexanone. Green Chem..

[23] Liu Y., Li G., Hu Y., Wang A., Lu F., Zou J.J., Zhang T. (2019). Integrated Conversion of Cellulose to High-Density Aviation Fuel. Joule.

[24] Wang W., Liu Y., Li N., Li G., Wang W., Wang A., Zhang T. (2017). Synthesis of renewable high-density fuel with isophorone. Sci. Rep..

[25] Guo J., Xu G., Han Z., Zhang Y., Fu Y., Guo Q. (2014). Selective Conversion of Furfural to Cyclopentanone with CuZnAl Catalysts. ACS Sustain. Chem. Eng..

[26] Hronec M., Fulajtárová K., Vávra I., Soták T., Dobročka E., Mičušík M. (2016). Carbon supported Pd–Cu catalysts for highly selective rearrangement of furfural to cyclopentanone. Appl. Catal. B Environ..

[27] Yang Y., Du Z., Huang Y., Lu F., Wang F., Gao J., Xu J. (2013). Yizheng Huang, Fang Lu, FengWang, Jin Gao and Jie Xu, Conversion of furfural into cyclopentanone over Ni–Cu bimetallic catalysts. Green Chem..

[28] Wang Y., Sun S., Li F., Cao X., Sun R. (2018). Production of vanillin from lignin: The relationship between β-O-4 linkages and vanillin yield. Ind. Crops Prod..

[29] Fache M., Boutevin B., Caillol S. (2016). Vanillin production from lignin and its use as a renewable chemical. ACS Sustain. Chem. Eng..

[30] Zhu Y., Liao Y., Lv W., Liu J., Song X., Chen L., Wang C., Ma L. (2020). Complementing Vanillin and Cellulose Production by Oxidation of Lignocellulose with Stirring Control. ACS Sustain. Chem. Eng..

[31] Camera-Roda G., Parrino F., Loddo V., Palmisano L. (2020). A Dialysis Photocatalytic Reactor for the Green Production of Vanillin. Catalysts.

[32] Klaus T., Seifert A., Häbe T., Nestl B.M., Hauer B. (2019). An Enzyme Cascade Synthesis of Vanillin. Catalysts.

[33] Gao H., Han F., Li G., Wang A., Cong Y., Li Z., Wang W., Li N. (2022). Synthesis of jet fuel range high-density polycycloalkanes with vanillin and cyclohexanone. Sustain. Energy Fuels.

[34] Zhang X., Song M., Liu J., Zhang Q., Chen L., Ma L. (2023). Synthesis of high density and low freezing point jet fuels range cycloalkanes with cyclopentanone and lignin-derived vanillins. J. Energy Chem..

