# Supplementary material for: Synthesis of Renewable High-Density Fuel with Vanillin and Cyclopentanone Derived from Hemicellulose
Source: Molecules. 2023 Jun 27;28(13):5029. doi: 10.3390/molecules28135029 (PMC10343900; doi:10.3390/molecules28135029)
Supplement: Supplementary file 1 [file molecules-28-05029-s001.zip › molecules-2398877-supplementary.pdf]

Synthesis of Renewable High-Density Fuel with Vanillin and Cyclopentanone Derived from Hemicellulose

Wei Wang[1], Ling An[1], Chi Qian[1], Yanqing Li[1], Meiping Li[2,*], Xianzhao Shao[1], Xiaohui Ji[1], Zhizhou Li[1]

1 Shaanxi Key Laboratory of Catalysis, School of Chemistry and Environment Science, Shaanxi University of Technology, No. 1 East One Ring Road Hanzhong 723001, China

2 College of Life Science, Shanxi University, No 92 Wucheng Road, Xiaodian District, Taiyuan 030006, China

* Correspondence: lmpmg@sxu.edu.cn

Figure S1. The ^{1}HNMR and ^{13}CNMR spectra of 2,5-bis(4-hydroxy-3-methoxybenzylidene)cyclopentanone……………………………………………………………2

Figure S2. Mass spectra of 2,5-bis(4-hydroxy-3-methoxybenzylidene)cyclopentanone……………3

Figure S3. The ^{1}HNMR and ^{13}CNMR spectra of 2,6-bis(4-hydroxy-3-methoxybenzylidene)cyclohexanone……………………………………………………………4

Figure S4. Mass spectra of 2,6-bis(4-hydroxy-3-methoxybenzylidene)cyclohexanone…………5

Figure S5. Gas chromatography of 2,5-bis (4-hydroxy-3-methoxybenzylidene) cyclopentanone hydrodeoxygenated product……………………………………………………………………5

Figure S6. Gas chromatography of 2,6-bis (4-hydroxy-3-methoxybenzylidene) cyclohexanone hydrodeoxygenated product……………………………………………………………………5

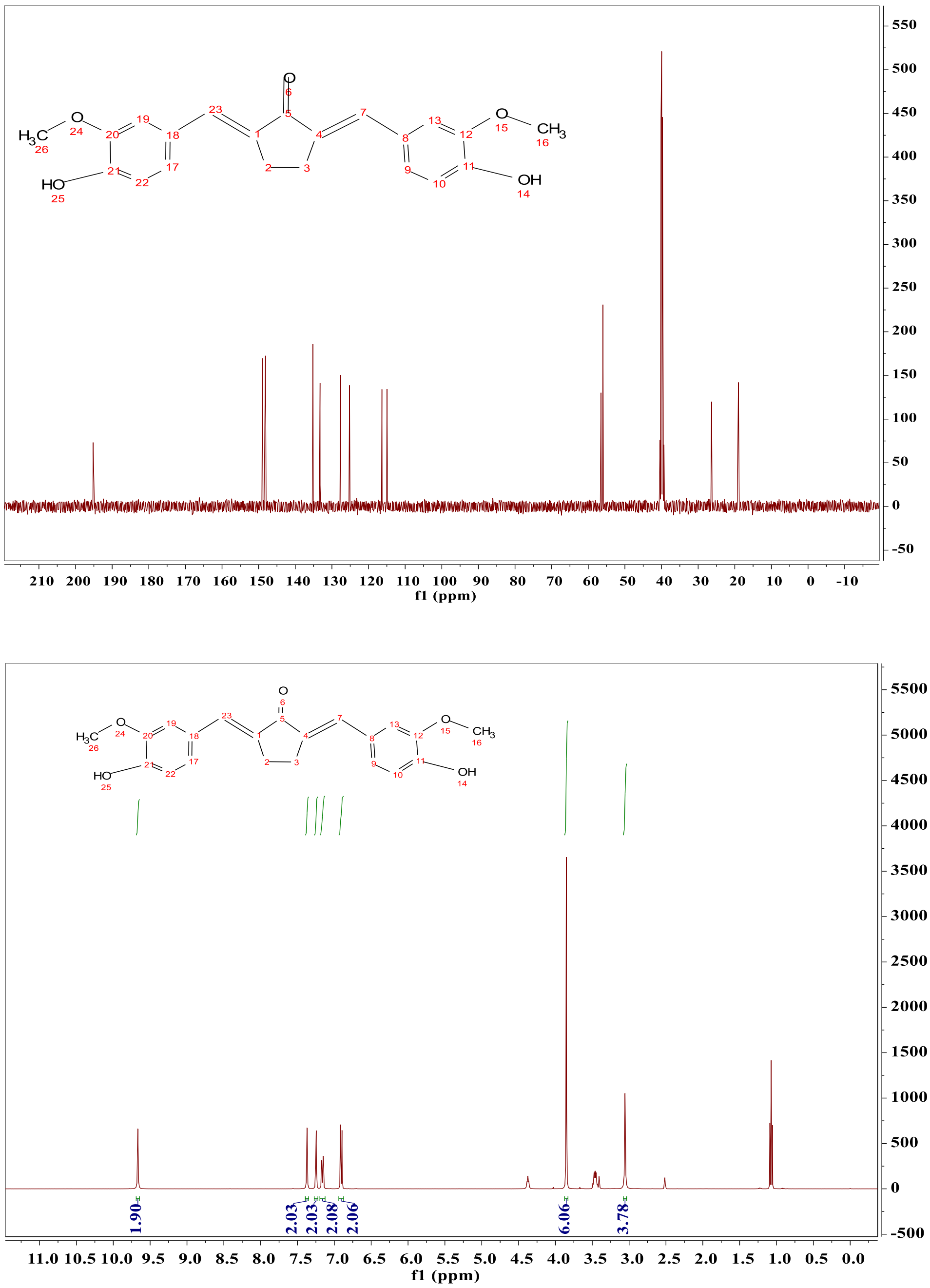

Figure S1. The ^{1}HNMR and ^{13}CNMR spectra of 2,5-bis(4-hydroxy-3-methoxybenzylidene)cyclopentanone

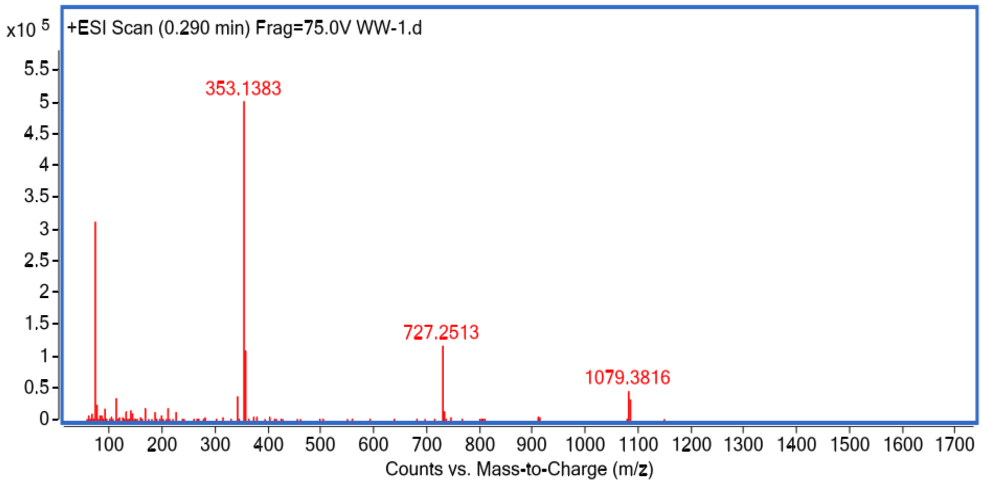

Figure S2. Mass spectra of 2,5-bis(4-hydroxy-3-methoxybenzylidene)cyclopentanone

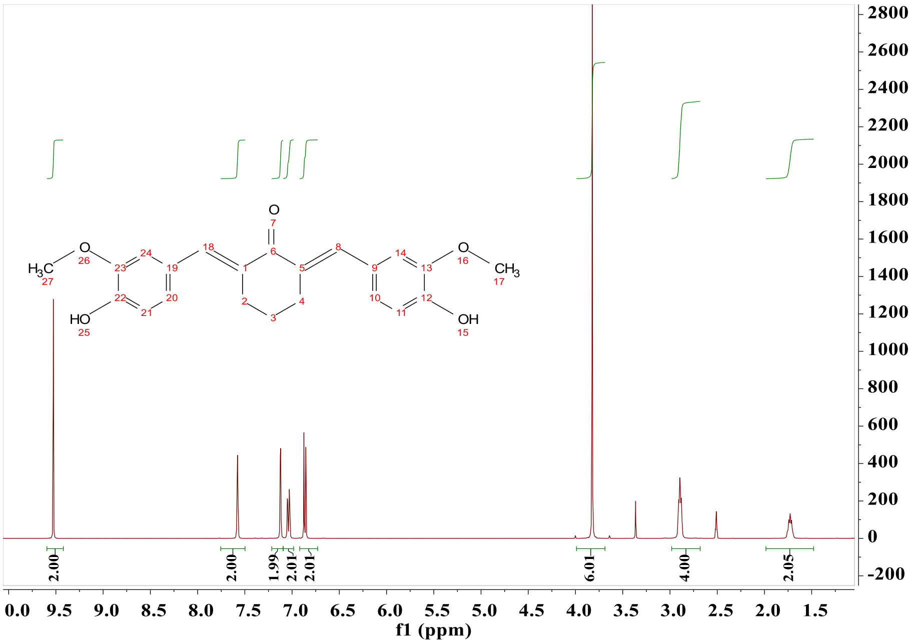

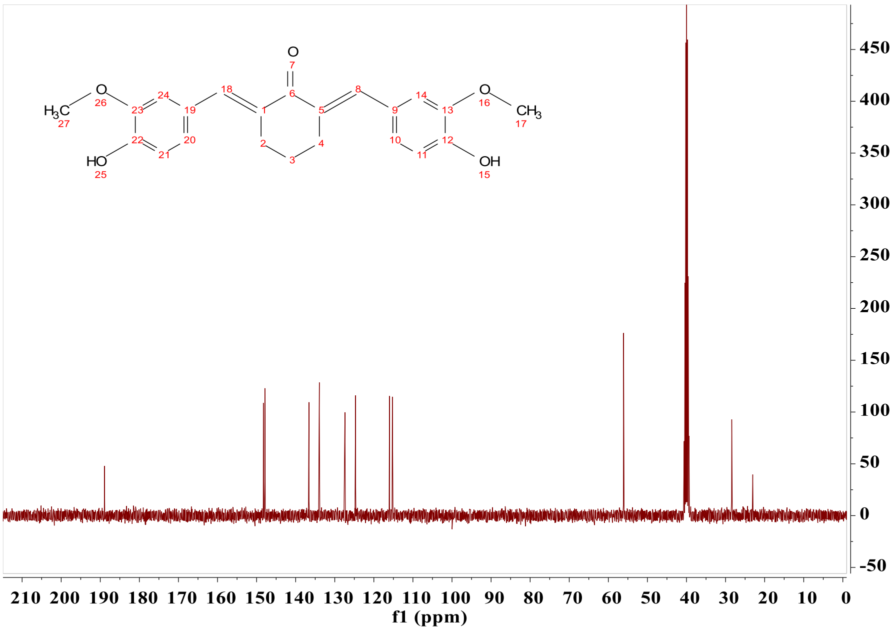

Figure S3. The ^{1}HNMR and ^{13}CNMR spectra of 2,6-bis(4-hydroxy-3-methoxybenzylidene)cyclohexanone

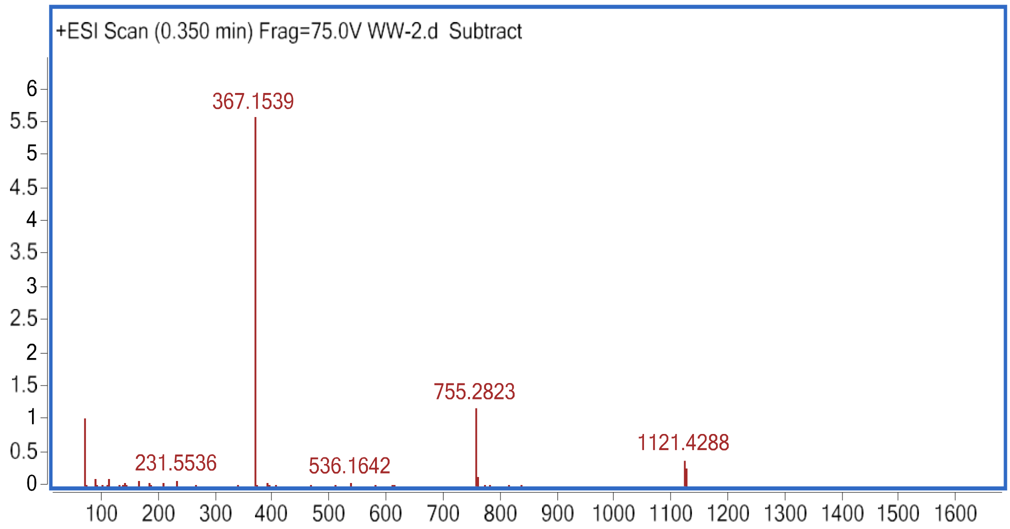

Figure S4 Mass spectra of 2,6-bis(4-hydroxy-3-methoxybenzylidene)cyclohexanone

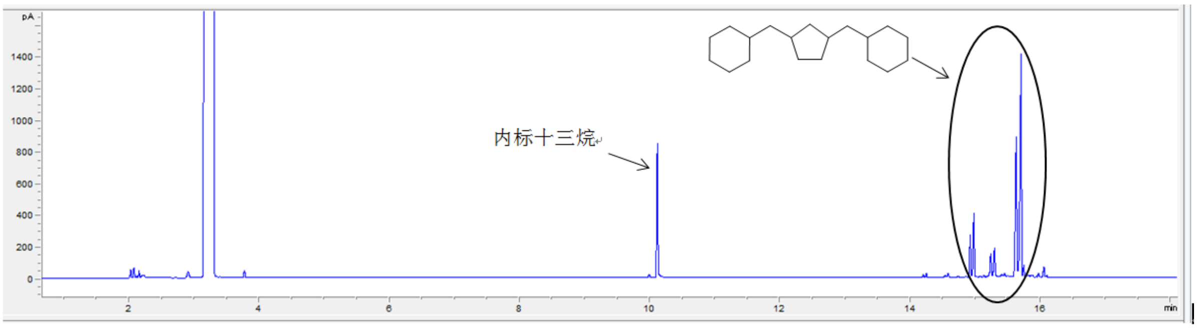

Figure S5. Gas chromatography of 2,5-bis (4-hydroxy-3-methoxybenzylidene) cyclopentanone hydrodeoxygenated product

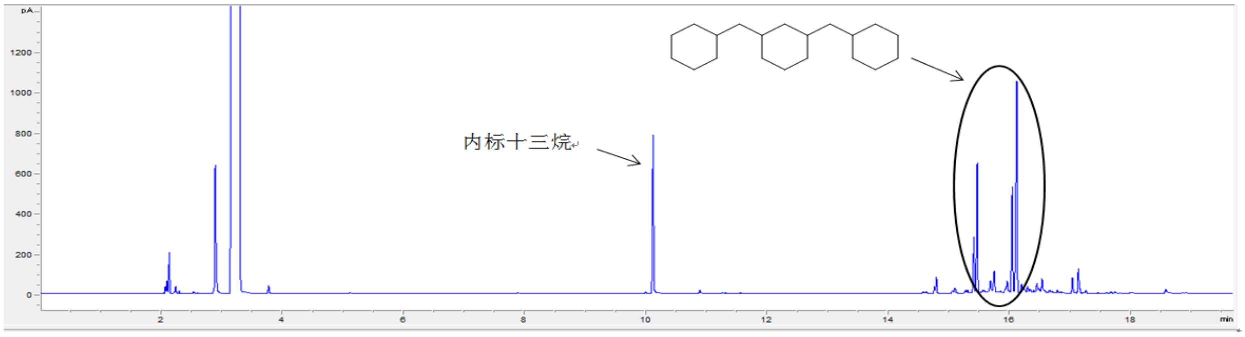

Figure S6. Gas chromatography of 2,6-bis (4-hydroxy-3-methoxybenzylidene) cyclohexanone hydrodeoxygenated product

Orthogonal experimental design was used to optimize reaction conditions, using a four factor three level orthogonal table as shown in Table S1. Nine experiments were conducted according to the parameters of the orthogonal table, and the experimental results are shown in Table S2.

Table S1. Factors and Levels

Serial Number	1	2	3	4
Factor name	Temperature	Catalyst dosage	Molar ratio of cyclopentanone to vanillin	Time
Level 1	60	0.075	1:1.5	6
Level 2	80	0.10	1:2.0	8
Level 3	100	0.15	1:2.5	10

Table S2 Orthogonal experimental results

Factor	T	C	M	t	Experimental result
Experiment1	1	1	1	1	53.1
Experiment2	1	2	2	2	62.5
Experiment3	1	3	3	3	65.2
Experiment4	2	1	2	3	82.5
Experiment5	2	2	3	1	83.5
Experiment6	2	3	1	2	68.6
Experiment7	3	1	3	2	81.7
Experiment8	3	2	1	3	63.7
Experiment9	3	3	2	1	82.5
Mean value 1	60.27	72.43	61.80	73.03	
mean value 2	78.20	69.90	75.83	70.93	
mean value 3	75.97	72.1	76.8	70.47	
range	17.93	2.53	15.00	2.57	

From the range value, it can be seen that the most significant factors affecting the reaction are temperature and the molar ratio of cyclopentanone to vanillin. The optimal reaction conditions are temperature 80 °C, molar ratio of cyclopentanone to vanillin 1:2.5, catalyst dosage 0.075g, and reaction time 6 hours. Conduct validation experiments using optimized conditions, parallel experiments three times, and the experimental results are 81.6, 82.1, and 82.6, respectively.
